# Characterization and Anti-Inflammatory Potential of an Exopolysaccharide from Submerged Mycelial Culture of *Schizophyllum commune*

**DOI:** 10.3389/fphar.2017.00252

**Published:** 2017-05-15

**Authors:** Bin Du, Yuedong Yang, Zhaoxiang Bian, Baojun Xu

**Affiliations:** ^1^Analysis and Testing Center, Hebei Normal University of Science and TechnologyQinhuangdao, China; ^2^School of Chinese Medicine, Hong Kong Baptist UniversityHong Kong, China; ^3^Food Science and Technology Program, Beijing Normal University–Hong Kong Baptist University United International CollegeZhuhai, China

**Keywords:** *Schizophyllum commune*, mycelium, exopolysaccharide, structure characterization, macrophage, anti-inflammatory activity

## Abstract

**Background and Purpose:** Mushroom polysaccharides have attracted attention in food and pharmacology fields because of their many biological activities. The structure characterization and anti-inflammatory activity of exopolysaccharide from *Schizophyllum commune* were evaluated in present study.

**Methods:** An exopolysaccharide from a submerged mycelial fermentation of *S. commune* was obtained using DEAE-52 cellulose and Sephadex G-150 chromatography. The molecular weight (MW), monosaccharide compositions, chemical compositions, methylation analysis, circular dichroism studies, Fourier transform infrared spectroscopy, nuclear magnetic resonance (NMR) spectra, scanning electron microscopy (SEM), and atomic force microscopy were investigated.

**Results:** It was a homogeneous protein-bound heteropolysaccharide with MW of 2,900 kDa. The exopolysaccharide contained a β-(1→3) glycosidic backbone, (1→4)- and (1→6)- glycosidic side chain, and high amount of glucose. The anti-inflammatory activity of exopolysaccharide was assessed by inhibiting the production of nitric oxide (NO), inducible nitric oxide synthase (iNOS), and 5- lipoxygenase (5-LOX) from macrophages. This exopolysaccharide significantly (*p* < 0.05) inhibited lipopolysaccharides-induced iNOS expression levels in the cells in a dose-dependent manner.

**Conclusion:** It indicated significant anti-inflammatory effects, which showed that exopolysaccharide might be exploited as an effective anti-inflammatory agent for application in NO-related disorders such as inflammation and cancer.

## Chemical compounds studied in this article

3-(4, 5-dimethylthiazol-2-yl-2, 5-diphenyltetrazolium bromide (PubChem CID: 16218671); carbazole (PubChem CID: 6854); trifluoroacetic acid (PubChem CID: 6422); ribose (PubChem CID: 5779); rhamnose (PubChem CID: 5460029); arabinose (PubChem CID: 66308); xylose (PubChem CID: 135191); mannose (PubChem CID: 18950); glucose (PubChem CID: 5793); and galactose (PubChem CID: 6036).

## Introduction

In the last few years there has been an upsurge of interest in mushroom polysaccharides that have been evaluated to be dietary fibers with medicinal effect (Giavasis, 2014). *Schizophyllum commune* (Fr.) is a species of basidiomycota belonging to *schizophyllaceae* of *agaricales* (Bae et al., 2012). It is one of the most widely distributed fleshy fungi, and can be isolated on all continents except for Antarctica (Teoh et al., 2012). *S. commune* is a filamentous growing fungus that produces exopolysaccharides (EPS). EPS isolated from a variety of micro-organisms are chemically well defined and have attracted worldwide attention due to their novel and unique physical and biological properties. These EPS have many industrial applications in food, pharmaceutical, and other industries as emulsifiers, stabilizers, binders, gelling agents, lubricants, and thickening agents (Jayakumar et al., 2010). Moreover, the most promising biological properties of these polysaccharides are their immune modulating and anti-cancer effects. A possible mechanism is that these polysaccharides are suggested to enhance cell-mediated immune responses *in vivo* and *in vitro* and act as biological response modifiers. Furthermore, the anti-inflammatory activities of polysaccharide also had been studied in these years. Du et al. (2015) reviewed the anti-inflammatory effects of fungal beta-glucan (a kind of polysaccharide). It has been found that a beta-glucan from *Alcaligenes faecalis* was capable of inducing IL-10-producing CD4 (+) T cells and inhibiting the development of eosinophilic airway inflammation (Kawashima et al., 2012). Moreover, Du et al. (2016) found that the anti-inflammatory activity of polysaccharide from *S. commune* was influenced by ultrasonic treatment. It has been proposed that the potent anti-inflammatory activity of polysaccharide, possibly due to the inhibition of pro-inflammatory cytokines or enhancing production of anti-inflammatory cytokines (Wang S. et al., 2014). The aim of current study was to characterize the *S. commune* exopolysaccharide by elemental analysis, high performance liquid chromatography (HPLC), gas chromatography (GC), methylation analysis, circular dichroism (CD) studies, Fourier transform infrared spectroscopy (FT-IR), nuclear magnetic resonance (NMR) spectra, scanning electron microscopy (SEM), and atomic force microscope (AFM). The anti-inflammatory effects of this exopolysaccharide were evaluated by determination of iNOS mRNA expression in RAW 264.7 macrophage cells and NO and 5-LOX production using enzyme-linked immunosorbent assay (ELISA).

## Materials and methods

### Materials and chemicals

Strains of *S. commune* were isolated from the fruiting bodies of wild mushroom *S. commune*. Dulbecco's Modified Eagle's Medium (DMEM) was purchased from Gibco-BRL (Gaithersburg, MD, U.S.A.). Fetal bovine serum (FBS), penicillin, streptomycin, carbazole, and 3-(4, 5-dimethylthiazol-2-yl-2, 5- diphenyltetrazolium bromide (MTT), *D*-glucuronic acid were obtained from Sigma-Aldrich (St. Louis, MO, U.S.A.). Ribose, rhamnose, arabinose, xylose, mannose, glucose, and galactose were purchased from Sinopharm Chemical Reagent Beijing Co., Ltd. (Beijing, China). DEAE-52 and Sephadex G-150 were obtained from the Pharmacia Co. (Sweden). All other chemicals were of analytical grade.

### Mycelial cultivation of *S. commune*

The mycelium of *S. commune* was cultivated on potato dextrose agar (PDA) slants. They were grown for 7 days at room temperature (28 ± 2°C) and non-contaminated slants were maintained and cultured. The culture was grown on PDA slants at 28 ± 2°C for 7 days. A 1 cm^2^ of mycelia along with agar from such slants were inoculated to 50 mL of sterile seed culture medium in 250 mL conical flasks, which were incubated at 28 ± 2°C, 180 rpm for 7 days on an orbit shaker (Kumari et al., 2008). Biomass concentration was determined by the dry mass method involving filtration of broth samples through pre-weighed filter discs (Whatman Ltd., Maidstone, UK). The filtrate was collected and stored at −20°C for the isolation of crude exopolysaccharides.

### Isolation and purification of exopolysaccharide from *S. commune*

The crude exopolysaccharides from *S. commune* was precipitated by overnight incubation with four-fold absolute ethanol. The exopolysaccharide precipitate was collected by centrifugation and de-proteinated by Sevage method (Miao et al., 2013). The crude exopolysaccharides were re-dissolved in distilled water and applied to a DEAE-52 column (2.7 × 70 cm). The column was eluted stepwise with distilled water, 0.1–1.0 M NaCl aqueous solution and fractions collected at a flow rate of 0.5 mL min^−1^ (each test tube hold 12 min) and monitored by the phenol-sulfuric acid method. Then the major fraction, named exopolysaccharide fraction a (peak a) (eluted by 1.0 M NaCl) was concentrated, dialyzed and lyophilized according to the elution curve. The exopolysaccharide was concentrated, lyophilized and further purified on a Sephadex G-150 column (2.6 × 60 cm). Each fraction was collected at a flow rate of 2 mL min^−1^ and measured total polysaccharide content by the phenol-sulfuric acid method at 490 nm and protein content at 280 nm by colorimetric method.

### Elemental analysis

Organic elemental microanalysis was carried out by Elementar Vario EL Instrument (Elementar, Germany) to analyze weight percentages of carbon (C), hydrogen (H), and nitrogen (N). All analyses were done in triplicate.

### Determination of uronic acid contents

The content of uronic acid in exopolysaccharide was determined using sulfuric acid-carbazole method. One milliliter of sample reacted with 5 mL 9.54 mg mL^−1^ of sodium tetraborate sulfuric acid solution in boiling water bath for 10 min, and then mixed with 0.2 mL 1.25 mg mL^−1^ of carbazole ethanol solution for another 10 min. The absorbance of the reaction solution was determined at 530 nm. *D*-glucuronic acid was served as a reference.

### Measurement of molecular weight

Exopolysaccharide was characterized for molecular weight using Agilent 1100 series HPLC system (Agilent Technologies Palo AHO, CA, U.S.A.) equipped with a TOSOH TSK-GEL G3000 SW XL column (7.8 mm × 30 cm, 10 μm; TOSOH Corp., Tokyo, Japan) and an refractive index detector. A sample of 20 μL was injected in the system by maintaining a flow rate of 0.5 mL min^−1^ and column temperature of 35°C. Separation was carried out using 0.05 mol L^−1^ phosphate buffer (pH 6.7) containing 0.05% NaN_3_ as mobile phase. The average molecular weight was calculated by the calibration curve obtained using various standard dextrans (738, 5,800, 11,220, 21,370, 41,800, 110,000, 118,600, 318,000, and 813,500; Ahmed et al., 2013).

### Monosaccharide composition analysis of exopolysaccharide

Monosaccharide compositions of exopolysaccharide were analyzed by GC as described by Liu et al. (2012) with some slight modifications. Briefly, 5 mg of exopolysaccharide was hydrolyzed with 4 mL 2 mol L^−1^ of trifluoroacetic acid (TFA) at 110°C for 2 h. After removing the residual TFA with methanol under reduced pressure, the sample was dissolved in 0.6 mL of pyridine and reacted with 10 mg of hydroxylamine hydrochloride and 2 mg of inositol (as internal reference) for 30 min at 90°C. Afterward, 0.8 mL of acetic anhydride was added and incubated for another 30 min at 90°C. Seven standard sugars (ribose, rhamnose, arabinose, xylose, mannose, glucose, and galactose) were converted to their acetylated derivatives according to the above-mentioned method. One microliter of sample derivatives was injected into Agilent 6890 N GC equipped with an HP-5 fused silica capillary column (30 m × 0.32 mm × 0.25 mm) and a flame ionization detector (FID). The oven temperature was maintained at 120°C for 3 min, and then increased gradually to 210°C at a rate of 3°C min^−1^. The relative molar proportions of sugars in exopolysaccharide were calculated by the area normalization method according to the chromatogram.

### Methylation and GC-MS analysis of exopolysaccharide

Methylation analysis of exopolysaccharide was carried out according to the published methods (Guo et al., 2008; Yin et al., 2012) with minor modifications. The dried exopolysaccharide was dissolved in anhydrous dimethyl sulphoxide, and then dry sodium hydroxide (30 mg) was added. The mixture was stirred for 3 h at 20°C, and then methyliodide was added into the mixture. The reaction was stopped by adding water. The methylated exopolysaccharides were then extracted with chloroform and then washed with distilled water for three times. The methylated products were further acetylated with acetic anhydride to obtain partially methylated alditol acetates. GC-MS analysis of exopolysaccharide was conducted on a DB-5 ms capillary column (0.25 μm × 0.25 μm × 30 m) using a temperature programing of 60–280°C at 5°C/min. Helium was used as the carrier gas. The components were identified by a combination of the main fragments in their mass spectra and relative GC retention times, comparing with the literature (Wang et al., 2007; Yin et al., 2012).

### Circular dichroism (CD) study of exopolysaccharide

The CD spectrum of the Congo Red-exopolysaccharide complex was measured in a Jasco model J-810 sepctropolarimeter by referring a literature (Ramesh and Tharanathan, 1998). Sample was scanned at 2 mg mL^−1^ concentration and specific ellipticity [θ] was calculated by the equation [θ] = *H* × *S/L* × *C*, where *H* is the height of the peak (cm), *S* is scale sensitivity, *L* is the path length, and *C* is the concentration (g mL^−1^).

### Measurement of infrared (IR) spectrum of exopolysaccharide

The IR spectrum of the exopolysaccharide was determined using a TENSOR 27 FT-IR spectrophotometer (Bruker Corporation, Karlsruhe, Germany). The sample was ground with spectroscopic grade KBr powder and then pressed into 1 mm pellets for FT-IR determination in the frequency range of 4,000 to 400 cm^−1^.

### NMR spectrum of exopolysaccharide

All NMR spectra were measured on a Bruker Avance III 600 NMR spectroscopy (Bruker Corporation, Karlsruhe, Germany). The samples were dissolved in D_2_O with 2,2-dimethyl-2-silapentane-5-sulfonic acid sodium salt (DSS), as an internal standard, while data were acquired at 298 K. Standard Bruker pulse sequences and usual processing parameters were used for heteronuclear singular quantum correlation (HSQC). Chemical shifts (δ) are expressed in ppm, coupling constants (*J*) in Hz.

### SEM analysis of exopolysaccharide

SEM technique was used for characterization of exopolysaccharide. Sample was fixed on aluminum stub and gold sputtered and examined through KYKY-2800 SEM (KYKY Technology Co., Ltd., Beijing, China) by maintaining an accelerated voltage of 10 kV.

### AFM of exopolysaccharide

The AFM of exopolysaccharide was carried out according to the report method with slight modifications (Wang et al., 2010). Exopolysaccharide solution (1 mg mL^−1^) was prepared with distilled water. The solution was continuously diluted to the final concentration of 0.1, 0.01 mg mL^−1^. About 5 μL of diluted exopolysaccharide solution was dropped on the surface of a mica sample carrier, and then absolute ethanol was drip on the sample to fix the exopolysaccharide. The mica carrier was scoured to remove the non-absorbed residue by double distilled water and subsequently allowed to dry at room temperature. Later, the AFM images were obtained by Agilent 5400 scanning probe microscope (Agilent Technologies, Palo Alto, CA, U.S.A.) in tapping mode. The cantilever oscillated at its proper frequency (158 kHz), and the driven amplitude was 0.430 V.

### Cell culture

RAW 264.7 murine macrophages were obtained from American Type Culture Collection (ATCC, Rockville, MD, U.S.A.). These cells were cultured at 37°C under 5% CO_2_-humidified air in Dulbecco's Modified Eagle's Medium (DMEM) supplemented with 10% fetal bovine serum (FBS), 100 U/mL penicillin, and 100 μg mL^−1^ streptomycin.

### Assay for cell viability

Cell viability was assessed using the MTT assay as described previously (Shin et al., 2008). In brief, RAW 264.7 cells were seeded into a 96-well plate at a density of 1.0 × 10^4^ cells per well and incubated at 37°C for 24 h. The cells were then treated with various concentrations of the samples. One hundred microliters of MTT (0.5 mg mL^−1^ in PBS) was added to the wells after additional 24 h incubation at 37°C, and the incubation continued for another 2 h. The resulting color was assayed at 540 nm using a microplate spectrophotometer (Molecular Devices, CA, U.S.A.).

### Determination of NO production

The nitrite concentration in the medium was measured by Griess reagent as an indicator of NO production as previously described (Shin et al., 2008; Li et al., 2014). Briefly, RAW 264.7 cells (1.0 × 10^5^ cells/well in a 24-well plate with 500 μL of culture medium) were pretreated with samples for 1 h and incubated for 16 h with LPS (100 ng mL^−1^). After incubation, the nitrite concentration in the supernatant (100 μL/well) was measured by adding 100 μL of Griess reagent. To quantify the nitrite concentration, standard nitrite solutions were prepared, and the absorbance of the mixtures was determined using a microplate spectrophotometer (Molecular Devices, CA, U.S.A.) at a wavelength of 540 nm.

### Determination of 5-LOX production

The inhibition of 5-LOX was measured using a colorimetric 5-LOX inhibitor screening kit (Cayman Chemical Co., Ann Arbor, MI, U.S.A.) according to the manufacturer's instructions.

### Real-time PCR analysis of iNOS mRNA

The real-time PCR was conducted to determine iNOS mRNA. Briefly, total RNA was isolated from RAW 264.7 cells using a TRIZOL reagent kit (Life technologies, Invitrogen, Carlsbad, CA, U.S.A.) according to the manufacturer's instructions. cDNA was synthesized using the SuperScript® First-Strand synthesis system for RT-PCR (Invitrogen, Carlsbad, CA, U.S.A.) under the manufacturer's instruction. Quantitative real-time PCR was performed on the ViiA™ 7 Real-Time PCR System (Applied Biosystems, Foster city, CA, U.S.A.) with Power SYBR GREEN Master Mix (Applied Biosystems, Foster city, CA, U.S.A.). The primer sequences for iNOS and beta-actin are as follows: iNOS, 5′-CACCTTGGAGTTCACCCAGT-3′ and 5′-ACCACTCGTACTTGGGATGC-3′; beta-actin, 5′-GGACAGTGAGGCCA GG ATGG-3′ and 5′-AGTGTGACGTTGACATCCGTAAAGA-3′.

### Statistical analysis

Analyses were performed in triplicate. Statistical analysis was performed using SPSS17.0 software package for Windows (SPSS Inc., Chicago, U.S.A.). Analysis of variance (ANOVA) was conducted, and Ducan's multiple range tests were used to determine the significant differences between groups measuring the probabilities of 0.05.

## Results and discussion

### Purification of exopolysaccharide from the mycelial culture of *S. commune*

**Figure 1:**
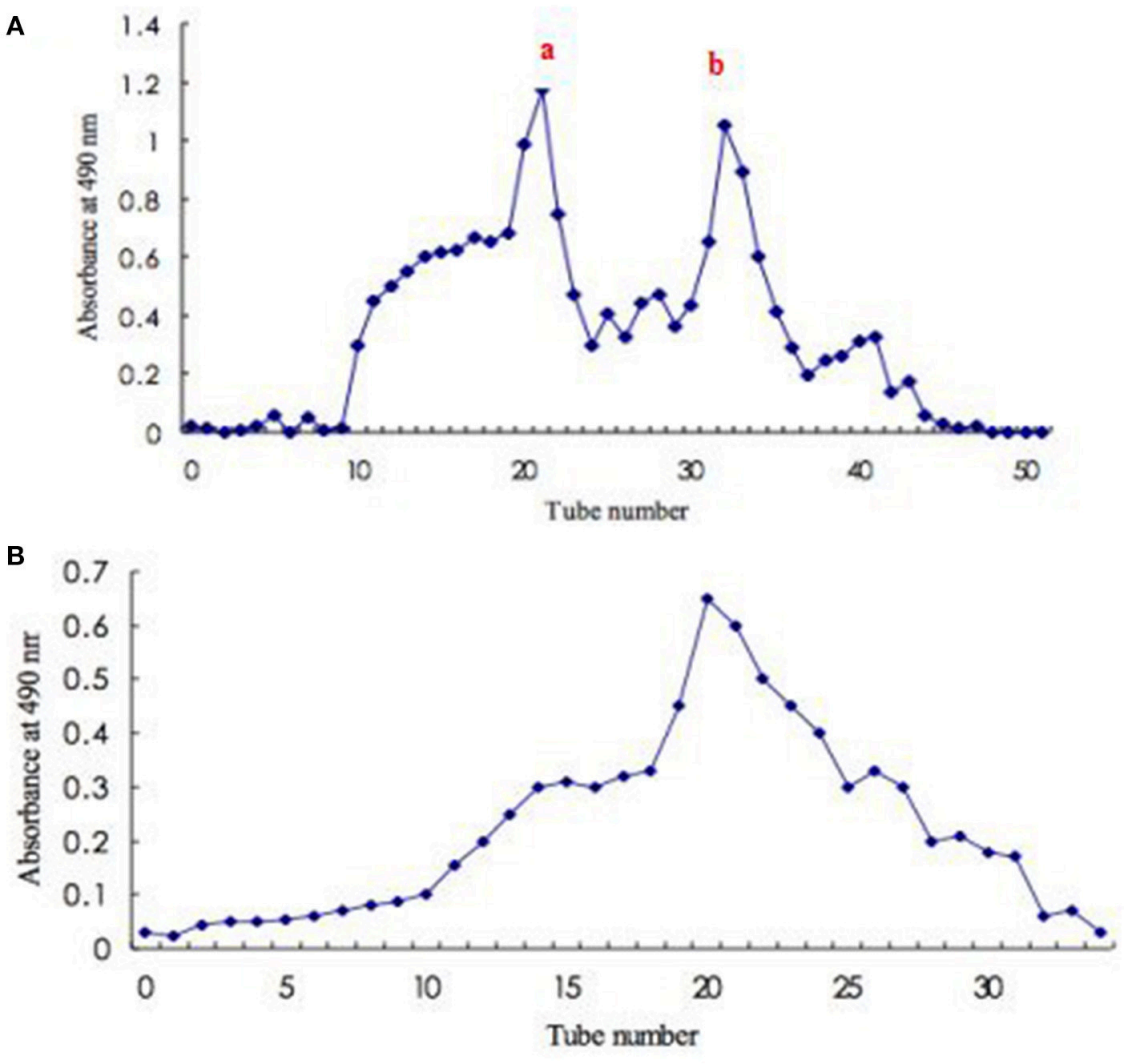
**Isolation and purification of exopolysaccharide from *S. commune***. **(A)** Elution profile (fraction a and fraction b) of crude polysaccharide on DEAE-52 anion-exchange column; **(B)** Elution profile to fraction a on Sephadex G-150 gel filtration column.

For exopolysaccharide production in submerged culture of *S. commun*e, there are some studies. Bolla et al. (2009) determined the effect of oils addition at different concentrations on the cell growth and production of exopolysaccharides in a submerged culture of *S. commune*. The results showed that the supplementation of the oils in the media substantially increased the exopolysaccharide production and 0.5% concentration proved to be ideal. Exopolysaccharide from the mycelial culture *S. commune* was firstly eluted through a DEAE-52 anion-exchange column to yield two peaks (fraction a and fraction b) (Figure 1A). Peak a (the main peak) was further purified by Sephadex G-150 gel filtration chromatography. Elution curve was shown as Figure 1B. Elution product was collected, dialyzed, and lyophilized to obtain an exopolysaccharide.

### Elemental analysis

The C, H, and N content (% w/w) of the exopolysaccharide was determined by elemental analysis. The elemental analysis of this exopolysaccharide gave the following results: C, 25.84%; H, 5.45%; and N, 0.65%. Usually, polysaccharide-rich samples do not contain nitrogen or show only small amounts of this element (up to 1%; Synytsya et al., 2009). The amount of nitrogen (% W/W) in exopolysaccharide obtained by elemental analysis is 0.65% in current study. Synytsya et al. (2009) proposed that nitrogen originated from proteins or from chitin. These proteins are probably bound to soluble glucans, and the Sevage method was not able to separate protein from protein-glucan complex completely. Results of elemental analysis were not in agreement with those published for aminated-derivatized exopolysaccharides (C, 37.94%; H, 5.84%; and N, 4.19%) from oat. It was then speculated that the different phenomena might depend on the different sources of polysaccharides: mushroom and oat.

### Chemical compositions of exopolysaccharide from the mycelial culture of *S. commune*

The carbohydrate content, protein content, and uronic acid content of exopolysaccharide were determined by colorimetric assays. The total carbohydrate content of exopolysaccharide was determined to be 89.0%, which presented high carbohydrate content. The content of protein was 2.2%, and this exopolysaccharide could be proved to be protein-bound polysaccharide because the Sevage method has been repeated many times to get rid of free proteins (Zhao et al., 2014). The uronic acid content of exopolysaccharide from the mycelial culture of *S. commune* was 7.52%. However, in the study of Klaus et al. (2011), no uronic acid was found in polysaccharides obtained from fruiting bodies of the wild mushroom *S. commune*. In current work, the exopolysaccharide was from mycelial culture of *S. commune*. Zhao et al. (2014) found that the uronic acid content of *plantago depressa* polysaccharide was 10.1%. In another study, the uronic acid content of a water soluble polysaccharide from fruiting bodies of *Agricus blazei* Murri was 5.5% (Dong et al., 2002). It can be speculated that the different uronic acid content obtained might depend on the natures of the materials.

### MW of isolated exopolysaccharides

**Figure 2:**
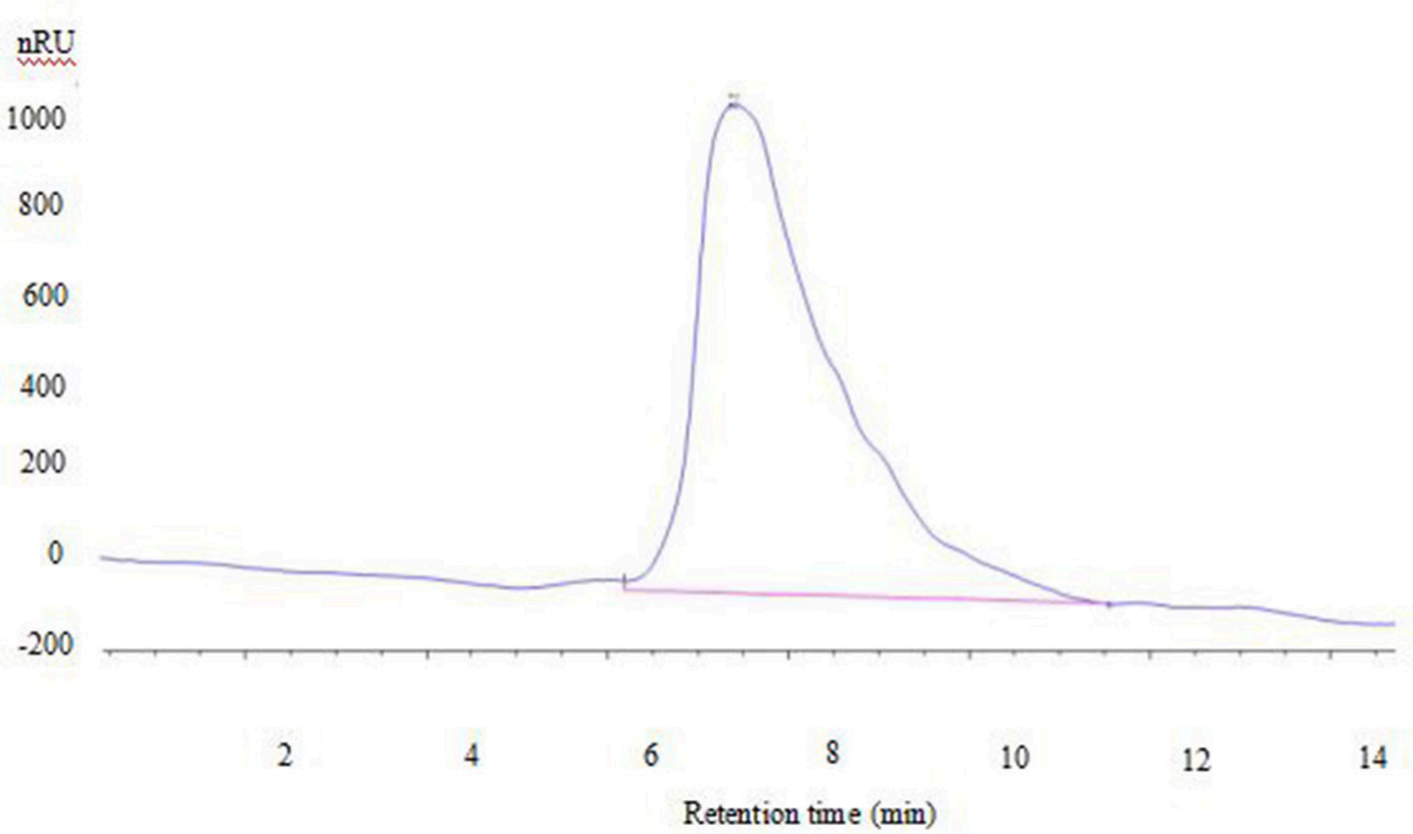
**GPC chromatogram of exopolysaccharide**.

Polysaccharide showed a single symmetrical peak in GPC profile, indicating it was a homogeneous polysaccharide (Figure 2). There is one peak in the chromatogram in aqueous solution, corresponding to the triple helix chains bounded with protein having high molecular weight. The MW of exopolysaccharide from *S. commune* was 2,900 KDa. In another studies, the MW of *S. commune*-derived β-glucan (MW = 1.5 × 10^5^ Da) produced by Mitsui Sugar Co., Ltd. (Tokyo, Japan) and a β-glucan purified from black yeast (*Aureobasidium* spp.) with MW of 1.78 × 10^5^ Da produced by Ace Biotech Ltd. (Cheongwon, Korea). The MW of the isolated polysaccharide was related with several factors, such as extraction temperature, the nature of the starting material, and fractionation methodology used (Ramesh and Tharanathan, 1998).

### Monosaccharide compositions of isolated exopolysaccharides

The monosaccharide compositions of exopolysaccharide were indicated in Table 1. The GC chromatogram of standard monosaccharides was shown in Supplemental Figure 1. The exopolysaccharide was a heteropolysaccharide and was composed of ribose, rhamnose, arabinose, xylose, mannose, glucose, galactose, and glucose was the main monosaccharide (57.5%). Some other reports presented the similar monosaccharide profiles as current polysaccharide. Klaus et al. (2011) suggested that presence of a large amount of glucose with smaller amounts of galactose and xylose.

**Table 1:** **Monosaccharide compositions in the exopolysaccharide from *S. commune***.

**Monosaccharides**	**Content (%)**
Ribose	3.79
Rhamnose	0.71
Arabinose	4.71
Xylose	1.93
Mannose	26.8
Glucose	57.5
Galactose	4.55

### Methylation analysis

The exopolysaccharide was methylated and measured by GC-MS in order to elucidate the linkages (Table 2). The GC-MS spectra of sugar residues after methylation reaction was indicated in Supplemental Figure 2. The terminals consisted of Ara (3.24%), Glc (3.17%), and Gal (0.47%), indicating polysaccharide was significantly branched and the side chains were terminated by the Ara residues. The high proportion of Ara residues suggested that some terminal Ara residues existed in the Ara side chains, and others were attached to the highly branched Gal side chains or connected to the back bone directly (Sun et al., 2010; Yin et al., 2012). The low proportion of terminal residues of Gal (0.47%) indicated that a part of the Gal side chains were terminated by the Ara residues.

**Table 2:** **GC-MS analysis for methylation of exopolysaccharide from *S. commune***.

PMAA^*^	**Relative percentage (%)**
1,2,3,4,5-Me_5_-Ara	3.24
1,3,4,5,6-Me_5_-Fru	0.14
1,2,3,4,5-Me_5_-Sor	0.18
2,3,4,5,6-Me_5_-Glc	3.17
1,2,3,4,5,6-Me_6_-Gal	0.47

* *PMAA, Partially O-methylated alditol acetates*.

### Circular dichroism analysis

**Figure 3:**
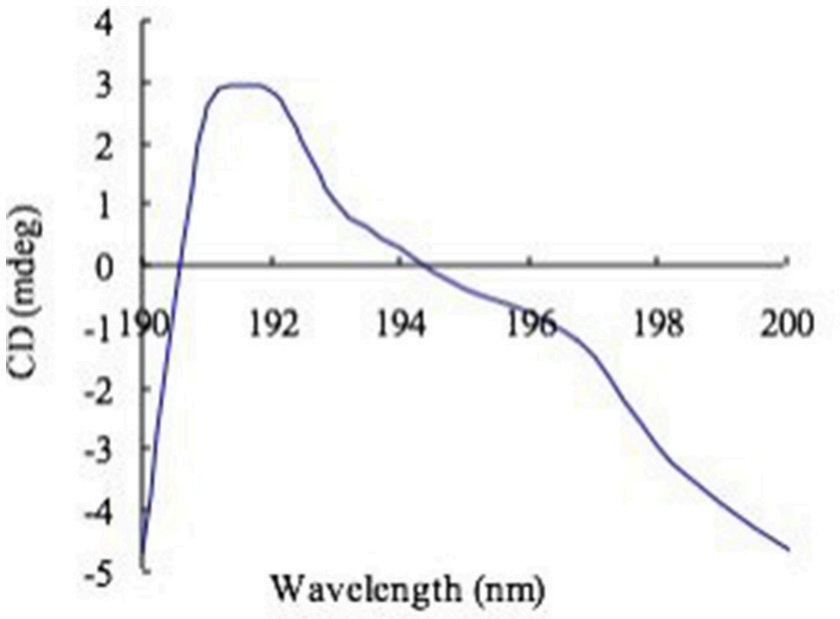
**CD spectra of Congo red complex with exopolysaccharide**.

CD study of the exopolysaccharide complex with Congo red (Figure 3) gave a positive band at 195 nm, which is exclusively composed of β-(1-3)-linkages. The CD spectral data were similar to those published polysaccharide (Ramesh and Tharanathan, 1998).

### The IR spectrum of isolated exopolysaccharide

The IR spectrum of isolated exopolysaccharide was shown in Supplemental Figure 3. The strong band at 3,386 cm^−1^ was assigned to the hydroxyl stretching vibration of the exopolysaccharide. The specific intense peaks at 2,934 cm^−1^ were due to the C-H stretching vibrations, respectively (Li et al., 2012). The high absorbency ranged of 1,082 cm^−1^ was the characteristic absorption peak of exopolysaccharide. This absorbance was attributed to the C-O-C and C-O-H link band. The exopolysaccharide exhibit characteristic absorption at 890 cm^−1^ for the β-configuration of glucan. It was in agreement with previously published result in which reported that polysaccharides showed an absorption band at 895 cm^−1^ assignable to the β- linkage, while two broad and intense bands and a shoulder appeared at about 1,065, 1,020, and 990 cm^−1^ (Robert et al., 2005). A band in the region of 1,611 cm^−1^ was due to associated water (Cao et al., 2006) and 1,723 cm^−1^ was C = O from uronic acids. The tiny signals at 2,362 and 1,611 cm^−1^ might be indicative of protein presence (N-H absorbing groups; Yang et al., 2012; Choma et al., 2013).

### Structural features of isolated exopolysaccharide

NMR spectroscopy is a powerful tool in structural analysis of fungal glucans (Synytsya and Novák, 2013). The ^1^H spectra (Supplemental Figure 4A) of exopolysaccharide indicated a chemical shift in the anomeric region at 4–6 ppm. Signal of 4.6 was obtained in current spectrum, corresponding to the chemical shift (ppm) of β-glucan (Gonzaga et al., 2005; Guerra Dore et al., 2007). The HSQC spectrum of anomeric region of exopolysaccharide at 298 K was shown in Supplemental Figure 4B. According to glucose anomeric structure, it is possible to distinguish α-*D*-glucans, β-*D*-glucans, and mixed α, β-*D*-glucans. By comparison of the spectra, the exopolysaccharide components are (1→3)—linked β-glucose or β-mannose (1→) (δ H-1 4.77) (Cho et al., 2008; Silveira et al., 2014). The signals in the non-sugar region (0.5–3 ppm for ^1^H) originate from the presence of proteins (Gonzaga et al., 2005). Signals in the ^13^C NMR spectrum of polysaccharide were assigned as much as possible according to literature values (Ramesh and Tharanathan, 1998; Yin et al., 2012). The anomeric signals in the ^13^C NMR spectrum of polysaccharide (Supplemental Figure 4C) were assigned partly according to correlations in the HSQC spectrum. ^13^C NMR spectrum of polysaccharide showed three signals at 72.46, 70.54, and 63.06 ppm corresponding to C-2, C-4, and C-6 nuclei, respectively. This indicates that (1→4)- and (1→6)-glucosidic side chain were found in this exopolysacchairde. It is well-known that (1→6)-β-glucosidic linkage is an important factor in influencing the biological activity, such as antitumoural effect (Borchers et al., 1999). The low-field chemical shifts indicated Ara residues were in furanose form and adopted α-anomeric configuration (Xu et al., 2010). It was in agreement with the results from methylation analysis.

### SEM analysis of isolated exopolysaccharide

**Figure 4:**
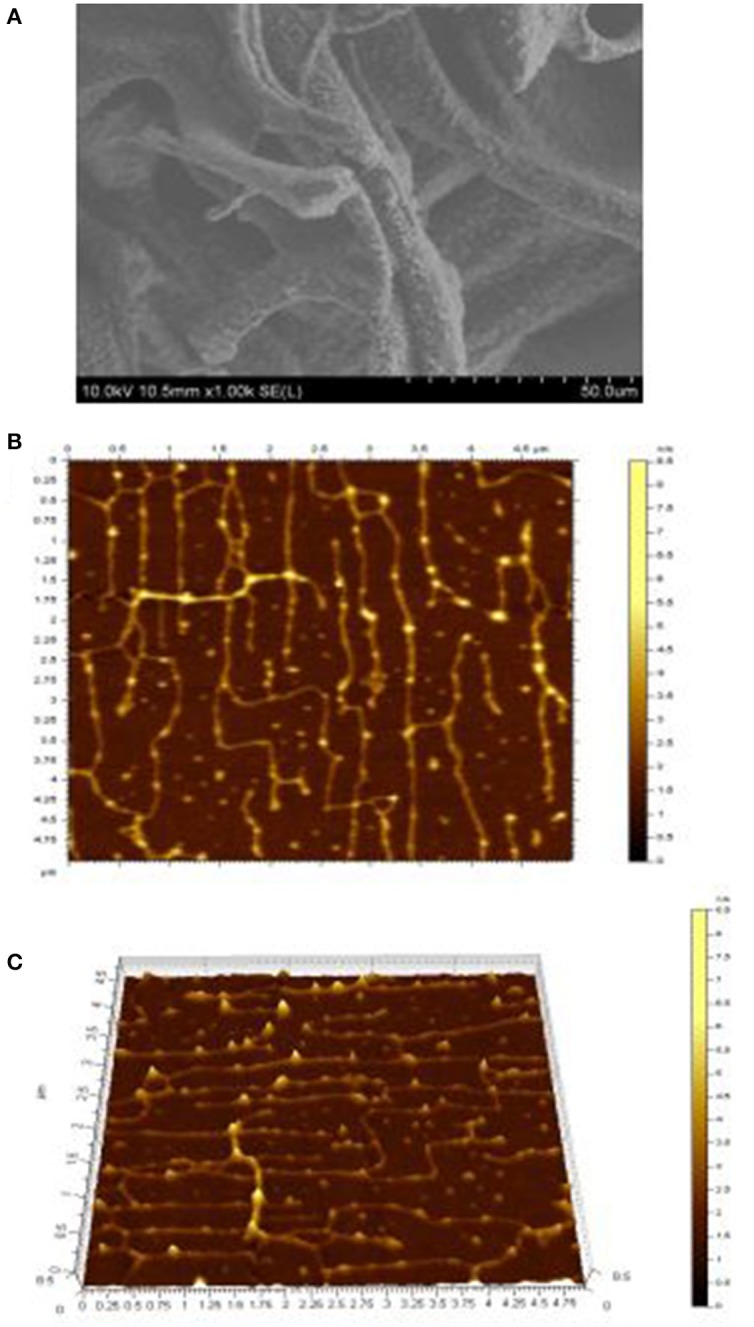
**Micrograph (A)** of exopolysaccharide assessed by SEM at 1,000 ×. Topographical AFM planar **(B)** and cubic **(C)** images of exopolysaccharide, the scan sizes are 1 × 1 μm for the images.

SEM is mostly used for imaging of exopolysaccharide and has been reported by many researchers (Goh et al., 2005) and as a very useful tool to study surface topography of polymers (Wang et al., 2010; Ahmed et al., 2013). SEM result of exopolysaccharide was indicated in Figure 4A. This exopolysaccharide looks like thin film with smooth and glittering surface. Furthermore, the SEM scan showed that the exopolysaccharide was made of a homogeneous matrix. Much of the SEM properties of exopolysaccharide are similar to the properties of polymer reported by Piermaria et al. (2008, 2009) and Ahmed et al. (2013), but was different from exopolysaccharide from *Lactobacillus plantarum* KF5 reported by Wang et al. (2010), in which the surface of polysaccharide was dull and had pores.

### Microstructure of isolated exopolysaccharide by AFM

Figure 4 depicts the topographical AFM planar (Figure 4B) and 3-dimensional images (Figure 4C) of exopolysaccharide. AFM is a powerful technique for directly observing the conformation of macromolecules under conditions that closely mimic the environments from which they originated (Han et al., 2011; Wang J. et al., 2014). It is clear from these images that the exopolysaccharide is linear and crossover in structure and branched or coiled in aqueous solution. The crystallite size and chain length of isolated exopolysaccharide in the current study were measured by AFM to be 3 nm and 1.6 μm, respectively (Table 3). Wang K. P. et al. (2014) investigated that the crystallite sizes of five bioactive polysaccharides from *Lentinus edodes* were 1.7–2.1 nm. They also observed in the morphology of the chains of the polysaccharide fractions, the sugar chains with high MW values were wider. They have hypothesized that hydrogen bonding led to the aggregation of the polysaccharide molecules, because the hydroxyl groups on the surface of the polysaccharides provided the strong intermolecular and intra-molecular interactions with each other.

**Table 3:** **Crystallite size and chain parameters of exopolysaccharide from *S. commune***.

Crystallite (nm)	3
Chain length (μm)	1.6
Point (nm)	1.56

### Effects of isolated exopolysaccharide on cell viability

To determine the cytotoxic effect of the exopolysaccharide on RAW 264.7 cells, the cells were treated with different concentrations (50–800 μg mL^−1^) of exopolysaccharide and incubated for 24 h. The exopolysaccharide did not affect cell viability at the concentration of 200 μg mL^−1^.

### Effects of isolated exopolysaccharide on LPS-induced NO, 5-LOX production and iNOS mRNA expression levels in RAW 264.7 cells

**Figure 5:**
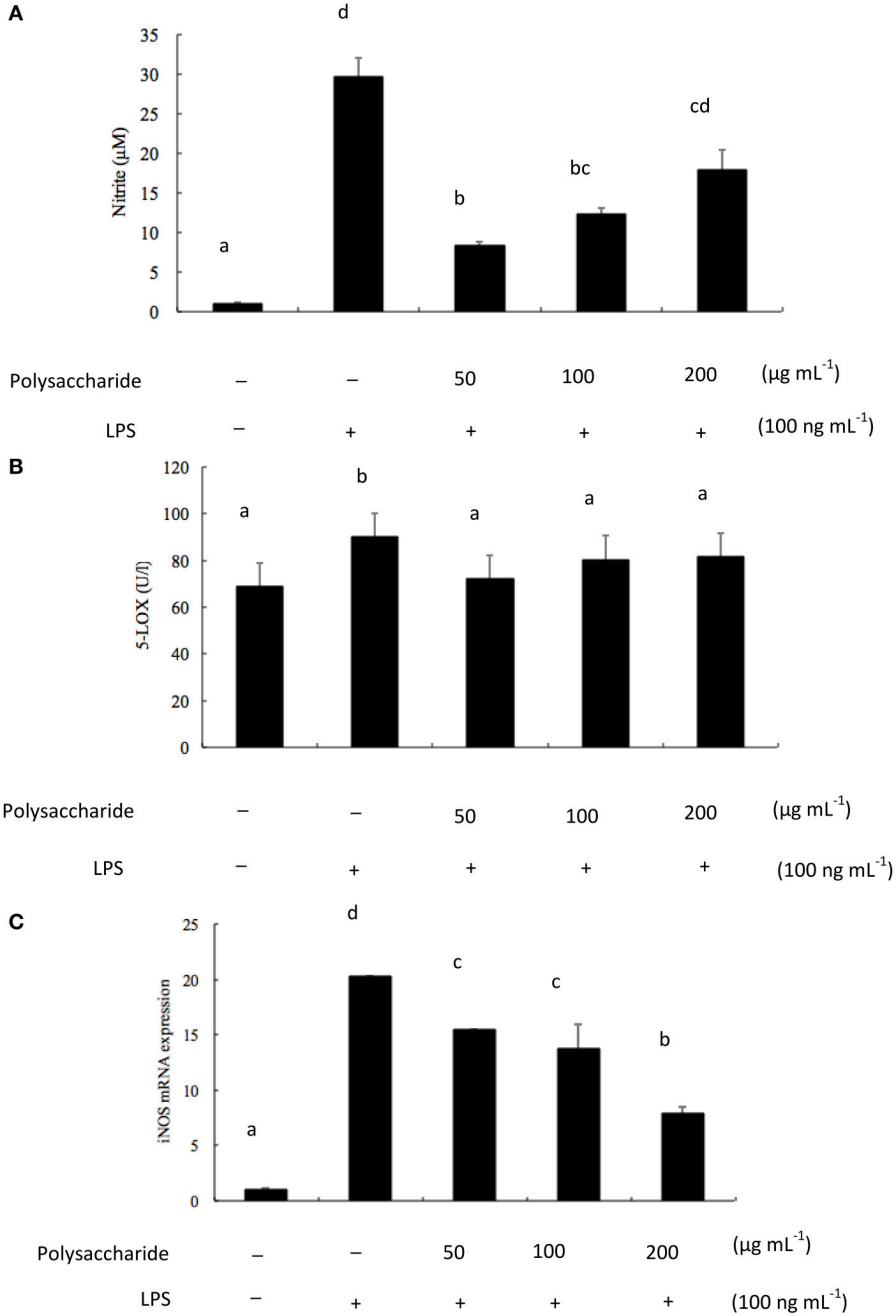
**(A)** Effects of exopolysaccharide on LPS-induced NO production. RAW 264.7 cells were pretreated with polysaccharide (50–200 μg mL^−1^) for 1 h before incubation with LPS (100 ng mL^−1^) for 16 h; **(B)** Effects of exopolysaccharide on LPS-induced 5-LOX production. RAW 264.7 cells were pretreated with polysaccharide (50–200 μg mL^−1^) for 1 h before incubation with LPS (100 ng mL^−1^) for 16 h; **(C)** Effects of exopolysaccharide on LPS-induced iNOS mRNA levels in RAW 264.7 cells. The cells were pretreated with the polysaccharide for 1 h before incubation with LPS (100 ng mL^−1^) for 16 h and total RNA was extracted. iNOS mRNA were measured by PR-PCR using specific primers. The bars labeled with the same letters are not significantly different at *p* < 0.05.

NO is recognized as a mediator and regulator in pathological reactions, especially in acute inflammatory responses (Surh et al., 2001). Since NO level is important in the evaluation of the extent of inflammation, the effects of exopolysaccharides on NO production was investigated. Pro-inflammatory agents, such as LPS, can significantly increase NO production in macrophages through activation of iNOS (Kojima et al., 2000). To determine the effects of exopolysaccharide on NO production, RAW 264.7 cells were incubated for 16 h with LPS (100 ng mL^−1^) in the presence of different concentrations (50–200 μg mL^−1^) of exopolysaccharide. Cell culture media were then collected and NO levels were measured. The exopolysaccharide inhibited NO production in LPS-induced RAW 264.7 cells (Figure 5A). Lee et al. (2009) found that *Agrocybe chxingu* polysaccharide suppressed NO production in a dose-dependent manner. However, it is not in agreement with some previously published results which showed that *Agaricus bisporus* polysaccharide stimulated NO production (Volman et al., 2010). Nandi et al. (2014) observed the enhanced production of NO in a dose-dependent of macrophages of β-glucan from edible mushroom *Russula albonigra*.

Moreover, 5-LOX is a key enzyme in the synthesis of leukotrienes, inflammatory mediators of arachidonic acid (Qu et al., 2000). RAW 264.7 cells were incubated for 16 h with LPS (100 ng mL^−1^) in the presence of different concentrations (50–200 μg mL^−1^) of exopolysaccharide. The effects of the exopolysaccharide on 5-LOX production were investigated. From Figure 5B, the exopolysaccharide decreased the production of 5-LOX, but not in a dose-dependent manner.

LPS is the main component of endotoxin, arrests macrophage proliferation, and activates macrophage to produce pro-inflammatory factors. NO is derived from the oxidation of *L*-arginine through three isoforms of nitric oxide synthase (NOS), namely neuronal (nNOS), and inducible (iNOS). Both iNOS and COX-2 are important enzyme mediators that mediate inflammatory processes (Wang et al., 2008). The current results indicated that the isolated exopolysaccharide inhibited LPS-induced iNOS mRNA expression levels in a dose-dependent manner (Figure 5C). Lee et al. (2009) suggested that a polysaccharide from *Agrocybe chaxingu* inhibited LPS-induced mRNA expression levels of iNOS and COX-2 in a dose-dependent manner. Moreover, Wang J. et al. (2014) reported the anti-inflammation activity of a water-insoluble β-(1→3)-*D*-glucan (derived from *Ganoderma lucidum*) against LPS induced RAW 264.7 cells. The RT-PCR results revealed the down- regulation of iNOS and TNF-α mRNA gene expression. However, Hashimoto et al. (1997) suggested that alkaline-treated schizophyllan (a kind of β-glucan from *S. commune*) was effective for iNOS production not only in isolated macrophages but also in tissue macrophages. It found that a single helical conformer is essential for iNOS production. The differences of polysaccharides in biological activities were probably due to their structural differences. In addition, conformation, monosaccharide compositions, linkage types, and molecular weight may affect their anti-inflammatory activities (Zhao et al., 2014). Further studies will be focused on the investigation of the relationship between polysaccharides structure and anti-inflammatory activity.

## Conclusion

In present work, an exopolysaccharide was isolated, purified and characterized from *S. commune*. This exopolysaccharide exhibited homogeneity with MW of 2,900 kDa and many evidences presented that it was bounded with protein, indicated as heteropolysaccharide. It belongs to a kind of β-(1→3)-D-glucans consisting of a backbone of β-(1→3)-linked glucose residues substituted with (1→4) and (1→6)-β-D-glucopyranosyl residues on main-chain residues. Monosaccharide composition analysis showed that the exopolysaccharide consisted with ribose, rhamnose, arabinose, xylose, mannose, glucose, galactose, and glucose was the main monosaccharide. The AFM images confirmed that the triple-helical chains of polysaccharide with high MW formed linear and crossover species. The triple-helical chains of polysaccharide exhibited the winding and bended shape rather than extended rigid state. The results indicated that the isolated exopolysaccharide could significantly decrease iNOS mRNA expression in a dose-dependent manner and NO and 5-LOX production from RAW 264.7 macrophages *in vitro*. These studies showed that *S. commune* exopolysaccharide had anti-inflammatory potential. Elucidation of anti-inflammatory mechanism in terms of signaling pathways on macrophages and animal model will be further studied.

## Author contributions

BX designed and funded the experiments; BD performed the experiments and wrote the manuscript; ZB designed and revised the manuscript; YY conducted part of experiments and analyzed the data.

## Funding

This research was jointly supported by two research grants (R201624 and R201627) from Beijing Normal University-Hong Kong Baptist University United International College, China.

### Conflict of interest statement

The authors declare that the research was conducted in the absence of any commercial or financial relationships that could be construed as a potential conflict of interest.
