# Supplementary material for: Characterization and Anti-Inflammatory Potential of an Exopolysaccharide from Submerged Mycelial Culture of Schizophyllum commune
Source: Front Pharmacol. 2017 May 15;8:252. doi: 10.3389/fphar.2017.00252 (PMC5430044; doi:10.3389/fphar.2017.00252)
Supplement: Supplementary file 1 [file DataSheet1.DOCX]

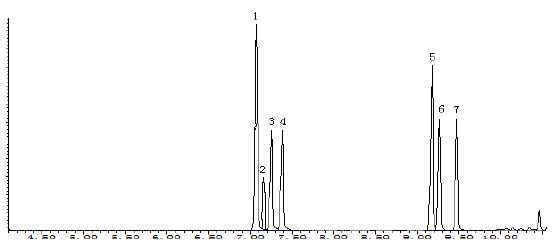


Abundance (%)

min

**Supplemental figure 1.** GC chromatogram of standard monosaccharides. 1: Ribose; 2: Rhamnose; 3: Arabinose; 4: Xylose: 5: Mannose; 6: Glucose; 7: Galactose.


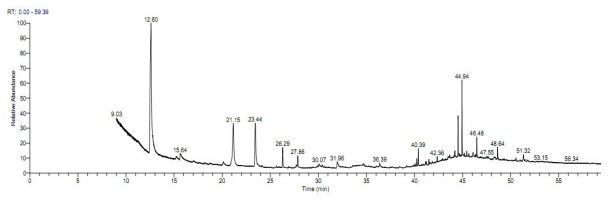


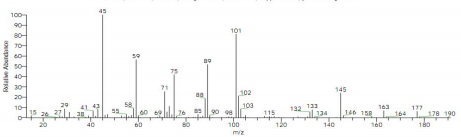


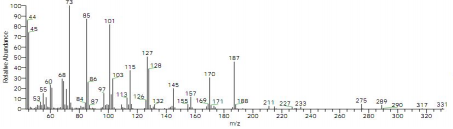

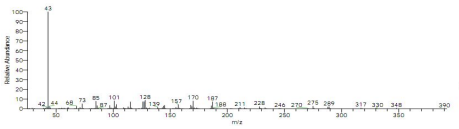

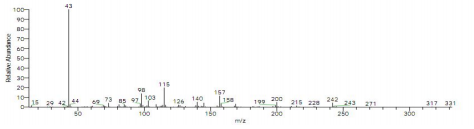

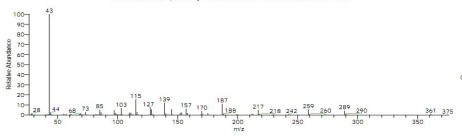


**Supplemental figure 2.** The GC-MS spectra of sugar residues after methylation reaction.


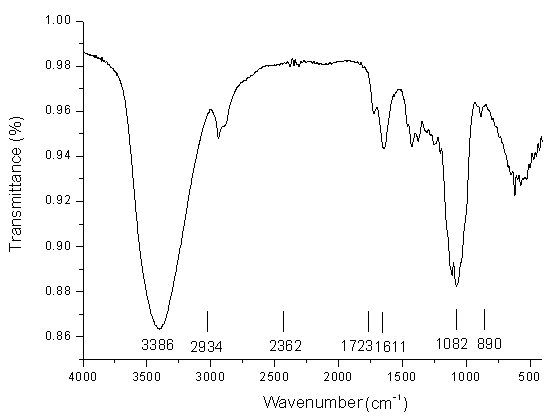


**Supplemental figure 3.** FT-IR spectrum of exopolysaccharide in the range of 4000-400 cm^-1^.

**(A)**


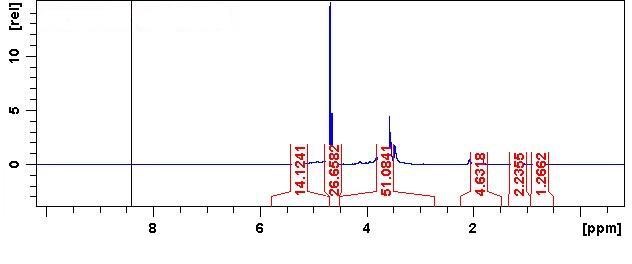


**(B)**


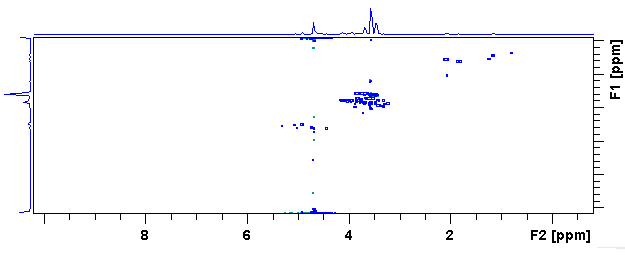
**
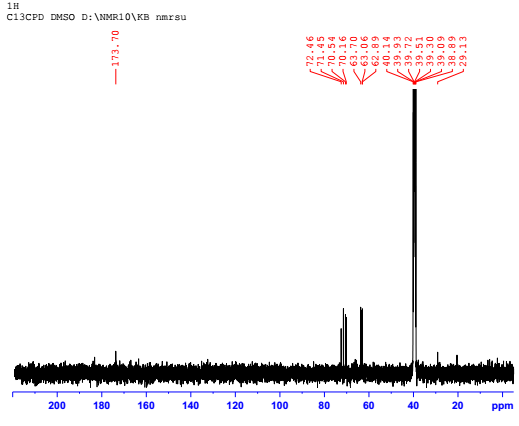
**

**(C)**

**Supplemental figure 4.** (A) The ^1^H-NMR spectra of exopolysaccharide measured at 298 K; (B) The HSQC spectrum of exopolysaccharide at 298 K; (C) The ^13^C NMR spectra of exopolysaccharide.
